# Medication and Healthcare Access on Medicare Costs Among ADRD Patients

**DOI:** 10.1002/alz70860_106018

**Published:** 2025-12-23

**Authors:** Jie Chen

**Affiliations:** ^1^ University of Maryland School of Public Health, College Park, MD, USA

## Abstract

**Background:**

This study examines the relationship between access to medication and healthcare with total Medicare costs, Part B costs, Part D costs, and out‐of‐pocket (OOP) prescription costs among Medicare fee‐for‐service (FFS) beneficiaries with ADRD.

**Method:**

This study utilizes linked data from the 2018, 2019, 2021, and 2022 Consumer Assessment of Healthcare Providers and Systems surveys and Medicare Beneficiary Summary Files. A cross‐sectional analysis was conducted using generalized linear models with a log‐normal and gamma distribution to account for the skewed distribution of costs. Survey weights were applied, and state fixed effects were included to control for geographic variations. Medication access was assessed using a self‐reported measure: “In the last six months, how often was it easy to get the medicines your doctor prescribed?”

**Result:**

The sample included 11,036 Medicare FFS beneficiaries aged 65+ with ADRD. The descriptive association between ease of obtaining medication and costs is nonlinear. Among FFS beneficiaries, those who reported that obtaining medication was “sometimes easy” had the highest average total Medicare costs ($40,518), followed by those who reported “usually easy” ($29,899), “always easy” ($26,391), and “never easy” ($22,992). After controlling for demographic characteristics, health needs, and geographic factors, findings indicate that easier access to prescription medication was associated with lower total Medicare costs (coef = ‐0.08, *p* = 0.001), lower Part D costs (coef = ‐0.20, *p* = 0.002), and lower Part D OOP costs (coef = ‐0.08, *p* = 0.019). However, access to prescription medication did not significantly impact Part B costs. In contrast, easier access to routine healthcare appointments was associated with higher Part B costs (coef = 0.183, *p* = 0.003) and higher Part B OOP costs (coef = 0.223, *p* = 0.002). ADRD FFS patients living in highly socially vulnerable areas experienced significantly lower Part B costs (coef=‐0.78, *p* = 0.001).

**Conclusion:**

Our findings highlight the importance of improving access to necessary medications for ADRD patients. Part B drugs, administered in physician offices, are crucial for dementia care, making access to routine care essential.

